# Comparison of sleep quality among COVID-19 patients and non-COVID-19 population in Pakistan: A cross sectional study during the COVID-19 pandemic

**DOI:** 10.1016/j.amsu.2022.103894

**Published:** 2022-06-04

**Authors:** Muna Malik, Ayesha Atiq, Muhammad Junaid Tahir, Fahd Kamal Akhtar, Muhammad Irfan Malik, Wardah Hassan, Fatima Muhammad Asad Khan, Iqra Akram, Noraiz Manhas, Irfan Ullah, Muhammad Sohaib Asghar

**Affiliations:** a Combined Military Hospital (CMH), Lahore, 54000, Pakistan; b Lahore General Hospital, Lahore, 54000, Pakistan; c Dow University of Health Sciences, Karachi, Pakistan; d Trakia University, Stara Zagora, Bulgaria; e Kabir Medical College, Gandhara University, Peshawar, Pakistan

**Keywords:** Sleep, COVID-19, SARS-CoV-2, Quality, Population

## Abstract

**Background:**

Adverse effects on the health and well-being changes may also express as a decreased sleep quality in COVID-19 patients. This study aimed to assess sleep quality among confirmed COVID-19 patients and the non-COVID-19 Pakistani population.

**Methods:**

An online cross-sectional survey was conducted between April and September 2020 in Punjab province, Pakistan. Information about demographic characteristics, COVID-19 diseased status, prior knowledge about COVID-19, and sleep quality using the Pittsburgh Sleep Quality Index (PSQI) was collected.

**Results:**

A total of 597 participants were included in this study, 296 (49.6%) COVID-19 patients and 301(50.4%) non-COVID-19 population. The PQSI was used to measure seven distinct sleep components; subjective quality, latency, duration, efficiency, disturbances, medication, and daytime dysfunction. Where mean ± standard deviation (SD) were 0.96 ± 0.743, 1.47 ± 1.032, 0.97 ± 1.006, 0.61 ± 0.995, 1.13 ± 0.649, 0.23 ± 0.651, 1.02 ± 0.861 respectively in total population (N = 597). Sleep latency, sleep duration, and sleep efficiency did not show a significant difference in the T-Test. While sleep quality, sleep disturbances, sleep medication, and daytime dysfunction showed a significant difference between both populations.

**Conclusion:**

In conclusion, we highlighted the poor sleep quality in COVID-19 patients as compared to the non-COVID-19 population**.**

## Highlights

• This study highlighted the poor sleep quality in COVID-19 patients as compared to the non-COVID-19 population.
• Sleep latency, sleep duration, and sleep efficiency did not show a significant difference.
• We found that Sleep quality, sleep disturbances, sleep medication, and daytime dysfunction components showed a significant difference.

## 1 Introduction

In December 2019, local health care services in Wuhan, China reported a group of patients suffering from undetermined pneumonia. Subsequently, the novel coronavirus was identified and was given the title of severe acute respiratory syndrome coronavirus 2 (SARS-CoV-2) [1]. Coronavirus disease 2019 (COVID-19) was also the label given to the disease by the World Health Organization (WHO), which additionally declared its outbreak on January 30, 2020 as a public health problem of international concern [2]. This rapidly progressive global pandemic has burdened many patients, mutually with physical and mental tribulations. Moreover, it is noted in patients with COVID-19 that their sleep quality is additionally disturbed with depression and anxiety that is accompanied by the illness [3]. A fundamental part of our physiological process is concomitant to sleep. It plays a vital role in regulating circadian rhythm and the hormone levels within the blood plasma that are powerful circadian constituents, such as melatonin and cortisol [4]. Therefore, in patients with COVID-19 sleep deficiency can lead to psychological and behavioral inabilities that largely affect academic performance simultaneously [5].

Moreover, the current situation of the pandemic not only disturbs the physical health but simultaneously affects the psychological wellbeing of the epidemic populace [6]. Historically, severe acute respiratory syndrome (SARS) in an epidemic setting has shown a potentiation in depression, stress, anxiety, sleep disturbances, and insomnia in a population [7]. Furthermore, in a pilot study, Richard et al. determined that trouble in sleep leads to certain maladaptive, hyperarousal, and anxiety-related behaviors that eventually advance to post-traumatic stress disorder (PTSD) [8].

Countries around the globe began the implementation of lockdown measures, which in turn led to significant social and lifestyle changes. Amongst these countries, Pakistan's government publicly announced its lockdown on March 14, 2020, in different provinces to address the perilous condition and to eventually diminish the infectivity of COVID-19 in the population [9]. Restrictions of the lockdown included the closure of universities, colleges, schools, shopping malls, and other areas of high social interaction, with the exceptional circumstances of leaving home for limited and approved purposes only [9]. In addition to this, living in quarantine throughout the pandemic affected the mental, psychological, and physical comfort of people to a great extent [10], thereby leading to disturbed daily routines, day-night sleeping patterns, and behaviors [11]. The transmission of COVID-19 is characteristically from close contact and droplet infection [12]. The recommendations of social distancing and isolation for COVID-19 disease prevention have exacerbated sleep disturbances and anxiety within the people following this preventive isolation [13].

Sleep also imparts an essential role in an individual's immunity, a vital modulator for immunity [14]. Deprived and modest sleep leads to a weakened immune system and increases the susceptibility of microorganisms such as viruses, bacteria, and parasites to cause infection [15]. This immunosuppression due to lessened sleep can be coupled with a deteriorating clinical picture of patients with COVID-19 [16]. A meta-analysis showed a 35% pooled prevalence of sleep disturbance during the COVID-19 pandemic [17], this will largely influence patients' immunity due to sleep deprivation during this pandemic.

There are many tools used in clinical and epidemiological prospects, to assess the presence of sleep disorders [18]. This study was aimed to assess sleep quality among the confirmed COVID-19 positive and non-COVID-19 Pakistani populations.

## 2 Methods

### 2.1 Participants and study design

The present cross-sectional web-based survey study was performed following the guidelines of observational studies in epidemiology. An online web-based Google survey was designed to collect the data in the English language during the first lockdown period from April to September 2020 for 6 months. This cross-sectional study was divided into two groups for online survey, one group included the population who were PCR confirmed cases of COVID-19 and the second group included those who were not infected with SARS-CoV-2 virus and were PCR negative for COVID-19 as recorded by the participants in the online survey. Both groups were invited through WhatsApp and social media groups irrespective of sociodemographic perspective. The inclusion criteria to participate in the study were being a Pakistani national residing in the Punjab province, having internet access, and voluntary participation. All the participants were randomly invited from contact lists of citizens by those who were collecting data through this online survey. The sample size required for this study was calculated using the Raosoft Sample size calculator with a 95% confidence interval, a 5% margin of error, and 50% response distribution. So, it was calculated that the minimum number of participants needed for this study was 377. A general rule of thumb is that a larger sample size will increase the generalizability of the results; therefore, we collected data for 6 months and tried to keep the sample size as large as possible. Incomplete submission of the survey questionnaire was not possible due to the implemented feature in the Google Forms that prevented submission of partially answered surveys; hence such responses were automatically excluded from the sample.

### 2.2 Measures

A structured closed-ended questionnaire in English was used for data collection. The questionnaire consisted of three sections: 1) demographics (consisting of basic information from participants such as gender, age, and profession), 2) COVID-19 diseased status, prior knowledge about COVID-19, and routine of outdoor activity during lockdowns, and 3) the Pittsburgh sleep quality index scale.

### 2.3 Pittsburgh sleep quality index (PSQI)

The PSQI is a commonly used self-reported scale to measure sleep quality for one month preceding the day of evaluation [19]. The scale consists of 19 items and cover seven component including subjective sleep quality, sleep latency, sleep duration, habitual sleep efficiency, sleep disturbances, use of sleep medication, and daytime dysfunction. The sum of seven components is called the PSQI global score. Each component is scored from 0 to 3 and the total score ranged between 0 and 21. A lower PSQI global score indicates better and good sleep quality. A global score of PSQI ≥5 is indicative of poor sleep quality. The PSQI is one of the most rigorously validated questionnaires to assess sleep quality. The PSQI has a very good diagnostic validity to characterize poor sleep quality with a sensitivity (89.6%) and specificity (86.5%) at the cut-off score of 5 [20].

### 2.4 Ethical consideration

The data collection was started after registering the study with the Ethical Review Committee of Lahore General Hospital (Unique identifying No (UIN): 00-136-20). Informed consent was obtained electronically from each participant at the start of the survey and the study was carried out following the Helsinki Declaration.

Data was gathered through messages using contacts list circle forwarding messages of Whatsapp, Facebook, and other social media platforms. As per the choice of participants, this online survey was voluntary and could be withdrawn from the survey at any moment.

### 2.5 Statistical analysis

Data were analyzed using SPSS 22.0 (IBM, Armonk, NY) for statistical analysis. Numerical variables are presented as mean and standard deviations; while, categorical variables are expressed as frequencies in percentages of two groups, i.e., COVID-19 patients and non-infected people. Chi-square and Fisher's Exact Test were applied to determine distribution differences in categorical variables. A global PSQI score was calculated based on the PSQI scoring manual. Independent sample T-Test was applied to calculate t-score and mean the difference between two groups, i.e., COVID-19 patients and non-infected people for all the components and the global PSQI scores. A dichotomous variable was generated based on the global PSQI score: those with a global PSQI score ≤5 were recorded as having good quality sleep, while those with a global PSQI score >5 were recorded as having poor sleep quality. A binary logistic regression analysis was performed with this dichotomous score (based on the PSQI global score) as the dependent variable, and demographic characteristics, COVID-19 diseased status, and any prior knowledge about COVID-19 as independent variables. *P*-value <0.05 was considered significant in all tests.

## 3 Results

A total of 597 participants were studied having an average age of 30.89 ± 10.32 years with a range of 16 years–75 years. A majority of the study sample (59.5%) belongs to the 21–30 years of age group; while, 24.8% belongs to 31–40 years of age. Of the total 597 participants, 296 (49.6%) participants were COVID-19 positive, and 301(50.4%) were not infected with the coronavirus at the time of the survey.

245 participants were females and 352 were males. Out of 245 females, 183 were infected with coronavirus, and 169 were not infected with that virus. Similarly, from 352 males, 113 males were infected with coronavirus, and 132 were non-infected population (Table 1).

**Table 1:** General demographics and sleep quality index (SQI).

		Population suffered with COVID-19 in % age n = 296		Population not suffered with COVID-19 in % age n = 301		p-Value
		Frequency (n)	Percent (%)	Frequency (n)	Percent (%)	
**Gender**	Male	113	38.2%	132	43.9%	**0.16**
	Female	183	61.8%	169	56.1%	
**Age Group**	<20 years	8	2.7%	12	4.0%	**<0.001**
	21–30 years	189	63.9%	166	55.1%	
	31–40 years	46	15.5%	102	33.9%	
	41–50 years	19	6.4%	11	3.7%	
	51–60 years	19	6.4%	6	2.0%	
	>60 years	15	5.1%	4	1.3%	
**Occupation**	Agriculture & Livestock	1	0.3%	0	0.0%	**<0.001**
	Banking & Financial Cooperative Sector	4	1.4%	5	1.7%	
	Businessmen & Entrepreneurs	12	4.1%	12	4.0%	
	Engineers	8	2.7%	24	8.0%	
	Labors	2	0.7%	0	0.0%	
	Judiciary & Lawyers	1	0.3%	3	1.0%	
	Law Enforcement & Security Forces	1	0.3%	1	0.3%	
	Media & Journalism	0	0.0%	2	0.7%	
	Healthcare Workers	221	74.6%	171	56.9%	
	Teachers	3	1.0%	27	9.0%	
	Housewives	23	7.8%	14	4.7%	
	Students	13	4.4%	31	10.3%	
	Govt. service	7	2.4%	11	3.7%	
**Education**	Uneducated	2	0.7%	0	0.0%	**<0.001**
	Secondary School & less	1	0.3%	2	0.7%	
	Higher Secondary Education	30	10.2%	36	12.0%	
	Bachelor	198	66.9%	151	50.2%	
	Masters & above	65	21.9%	112	37.1%	
The routine of going outdoor during this Lock-down in the last 2 weeks	0 time, observing full-time stay-home policy.	47	15.9%	72	23.9%	**<0.001**
	1- 2 times	102	34.5%	95	31.6%	
	3 - 5 times	58	19.6%	49	16.3%	
	>5 times but not daily	89	30.1%	34	11.3%	
	Daily	0	0.0%	51	16.9%	
Subjective Sleep Quality (Component 1 SQI)	Very good	59	19.9%	94	31.2%	**0.01**
	Fairly good	177	59.8%	162	53.8%	
	Fairly bad	45	15.2%	36	12.0%	
	Very bad	15	5.1%	9	3.0%	
Calculated Sleep Quality PSQI Scores	Good (PSQI ≤5)	114	38.5%	155	51.5%	**<0.001**
	Bad (PSQI >5)	182	61.5%	146	48.5%	

Seven components of PSQI were calculated in both COVID-19 positive (N = 296) and negative (N = 301) population. The components of sleep calculated were Subjective quality, latency, duration, efficiency, disturbances, medication and day time dysfunction; mean ± standard deviation (SD) were in total population (N = 597) 0.96 ± 0.743, 1.47 ± 1.032, 0.97 ± 1.006, 0.61 ± 0.995, 1.13 ± 0.649, 0.23 ± 0.651, 1.02 ± 0.861 respectively, these scores summed up to calculate global PSQI scores; which were for total population (N = 597) 6.38 ± 3.620, COVID-19 Positive (N = 296) 6.96 ± 3.770 and COVID Negative (N = 301) 5.82 ± 3.378 (See Table 2).

**Table 2:** Distribution of how both populations rated their subjective sleep quality overall for the last one month in this Pandemic (From component 1 of PSQI).

Component		Means			Levene's Test		*t*-test for Equality of Means				
		Total Population (N = 597)	COVID Positive (N = 296)	COVID Negative (N = 301)	F	p-Value	t	p-Value	Mean Difference	95% Confidence Interval	
										Lower	Upper
**1**	**Subjective sleep quality**	0.96 ±0.74	1.05 ±0.74	0.87 ± 0.73	1.72 ^	0.193	3.096 df 595	<.01	0.187 **	0.068	0.306
**2**	**Sleep latency**	1.47 ±1.03	1.51 ±1.03	1.42 ± 1.03	0.01 ^	0.91	1.124 df 595	0.26	0.095 ^	−0.071	0.261
**3**	**Sleep duration**	0.97 ±1.01	1.05 ±1.02	0.89 ± 0.99	0.09 ^	0.77	1.951 df 595	0.05	0.160 ^	−0.001	0.322
**4**	**Habitual sleep efficiency**	0.61 ±1.00	0.67 ±1.03	0.55 ± 0.96	4.41 *	0.04	1.442 df 590.050	0.15	0.117 ^	−0.042	0.277
**5**	**Step disturbances**	1.13 ±0.64	1.23 ±0.70	1.04 ± 0.58	22.38 **	0.00	3.472 df 570.120	0.001	0.183**	0.080	0.287
**6**	**Use of Sleep medication**	0.23 ±0.65	0.28 ±0.74	0.17 ± 0.54	16.54 **	0.00	2.021 df 541.698	0.04	0.108*	0.003	0.212
**7**	**Daytime dysfunction**	1.02 ±0.86	1.16 ±0.90	0.87 ± 0.808	5.18*	0.02	4.143 df 584.437	<0.001	0.288 ***	0.152	0.425
Global PSQI Score		6.38 ±3.620	6.96 ±3.770	5.82 ± 3.38	2.57 ^	0.11	3.889 df 595	<0.001	1.139 ***	0.564	1.714
**Levene's Test for Equality of Variances** ^ p > 0.05 Equal variances assumed *p < 0.05 Equal variances not assumed **p < 0.001 Equal variances not assumed								^ p > 0.05 *p < 0.05 **p < 0.01 ***p < 0.001			

Sleep latency, sleep duration, and sleep efficiency did not show up a significant difference in the T-Test. While sleep quality, sleep disturbances, sleep medication, and daytime dysfunction showed a significant difference between both populations. Global PSQI scores were having a mean difference of 1.139 which is highly significant (p < 0.001). Female gender, level of education and COVID-19 infection was associated with PSQI >5 (Table 3).

**Table 3:** Odds Ratio for poor Sleep Quality (PSQI >5).

Risk Factor		Odds Ratio	95% Confidence Interval		p-Value
			Lower	Upper	
Age	≤28 years	1.29	0.94	1.78	0.12
	>28 years	Reference group			
Gender	Male	Reference group			<0.001
	Female	1.84	1.32	2.55	
Education	≤ Bachelor	1.59	1.11	2.26	0.01
	> Bachelor	Reference group			
Profession Healthcare Workers	Yes	1.18	0.84	1.66	0.33
	No	Reference group			
COVID-19 Infection	Yes	1.70	1.22	2.35	0.001
	No	Reference group			
COVID-19 Knowledge (Subjective)	Yes	Reference group			0.68
	No	1.64	0.15	18.18	

## 4 Discussion

Of 296 COVID-19 patients assessed for sleep quality during their illness 182 (61.5%) suffered from poor sleep quality (PSQI>5) as compared to the non-COVID-19 infected population. These findings are in line with the latest study conducted by Akinci et al. [21] on 189 patients and can be justified by the fact that home isolation after getting infected with coronavirus is largely leading towards deleterious physical and mental health challenges [22]. The rapid increase in the levels of stress and anxiety after getting infected with the virus leads to sudden activation of the hypothalamic-pituitary-adrenal axis (HPA axis), and potentiation of a continuous cycle of insomnia and stress [23], thereby compromising overall sleep quality and sound mental health [24]. In addition to that, the HPA axis activation and insomnia lead to an abnormal upsurge in cortisol levels which suppresses the number and activity of natural killer (NK) cells, thereby imposing negative effects on the efficiency of the immune system [25]. Furthermore, circadian rhythm also plays a vital role in maintaining the immune system. The differentiation of progenitor cells to macrophages and secretion of melatonin from cells are carefully synchronized during nighttime sleep [20]. Disturbance in the rhythm due to suboptimal quality of sleep and stress decreases the activity and production of immune cells [26] and the overall ability of the immune system to fight against the infection effectively.

Moreover, our analysis concluded that there is a significant difference between the global PSQI scores in the components of daytime dysfunction, subjective sleep quality, sleep disturbances, and sleep disturbances individuals that tested positive for COVID-19 compared to those who did not, indicating a relative decrement in the sleep quality of the infected population. This is consistent with the results of a cohort study that reported sleep disturbances among the primary long-term health consequences of COVID-19 infection in 1733 adult participants from Wuhan, China [27]. Although the exact mechanism through which viruses affect sleep modulation remains unclear, these findings may be attributed to direct injury to CNS structures by the pathogen, or its subsequent activation of the immune response and cytokine production which alters the sleep centers in the brain [28].

In agreement with our findings, cross-sectional studies conducted by Marelli et al. [29] and Alharbi et al. [30] showed that the female gender was a significant risk factor for poorer sleep quality. A recent systematic review evaluating the impact of COVID-19 on mental health showed that women were more prone to psychological distress and psychiatric symptoms which could explain the lack of sleep efficiency [31]. Similarly, Alharbi et al. [30] identified primary education status as another risk factor in their analysis of 790 individuals, which corresponds to our results where people without a bachelor's degree of qualification were more likely to score higher on the PSQI. Moreover, no association between age and sleep quality was depicted which is confirmed by our results. However, female patients significantly had a poor sleep quality as compared to male patients possibly due to higher psychological stress in females and partially due to their work being more doubled by COVID-19 and the care burden in-home [32].

In Madrid, Spain, Herrero et al. compared sleep characteristics of 170 healthcare professionals and non-healthcare workers and reported that 64% of people employed in the healthcare sector had poor sleep quality in contrast to 44% in the non-healthcare worker group [33]. Likewise, Huang and Zhao also estimated the risk of poor sleep quality to be higher in those associated with the healthcare profession in their cross-sectional analysis [34]. This is in contrast to our findings which do not indicate a significant connection between sleep quality and medical occupation. However, these discrepant results may be explained by the varying study times. The aforementioned studies were conducted in early 2020 when the medical community was struggling to grapple with the threat of the novel coronavirus amid a surge of cases, precipitating increased workload and psychological stress on frontline workers.

This study had some limitations. Firstly, due to the cross-sectional design of our study, it was difficult to create casual interventions. Secondly, the nature of sleep quality of patients was not assessed before their disease due to sudden unexpected occurrence of COVID-19 infection. Thirdly, we used an online questionnaire instead of a one-to-one interview-based technique that may have contributed to the addition of bias due to self-reporting outcomes in the results. Lastly, non-subjective sleep evaluation methods such as polysomnography are required to further enhance the reliability of the results.

## 5 Conclusion

In conclusion, we highlighted the poor sleep quality in COVID-19 patients as compared to the non-COVID-19 population. Any change in sleep quality affects mental health and the capability of the body to cope with serious infections like COVID-19. Therefore, important mental health counseling and awareness sessions should be regularly practiced by healthcare professionals while dealing with COVID-19 patients to ensure proper sleep quality and enhancement in the immune system among the patients thereby decreasing the overall burden on intensive care units (ICUs) and healthcare.

## Ethical approval

Ethical approval was taken in this study from institutional review board of Lahore General Hospital (Ref no: 00-136-20)

## Sources of funding for your research

None.

## Author contribution

M.M, F·K.A, and M.I.M conceived the idea; A.A, M.M, F.K.A, M.I.M, and I.A collected the data; F.K.A and I.U analyzed and interpreted the data; M.M, W.H, M.J.T, F.M.A.K, I.U, and N.M did write up of the manuscript; and finally, I.U, M.J.T, M.S.A and F.K.A reviewed and revised the manuscript for intellectual content critically. All authors approved the final version of the manuscript.

## Conflicts of interest

None.

## Registration of research studies

1. Name of the registry: Lahore General Hospital
2. Unique Identifying number or registration ID: 00-136-20.
3. Hyperlink to your specific registration (must be publicly accessible and will be checked):

## Guarantor

Muhammad Sohaib Asghar.

## Consent

Informed consent was obtained electronically from each participant at the start of the survey.

## Provenance and peer review

Externally peer reviewed, not commissioned.

## Funding

None.

## Ethics statement

All ethical requirements were fulfilled before commencement of study.
